# Towards *in cellulo* virus crystallography

**DOI:** 10.1038/s41598-018-21693-3

**Published:** 2018-02-28

**Authors:** Helen M. E. Duyvesteyn, Helen M. Ginn, Maija K. Pietilä, Armin Wagner, Johan Hattne, Jonathan M. Grimes, Elina Hirvonen, Gwyndaf Evans, Marie-Laure Parsy, Nicholas K. Sauter, Aaron S. Brewster, Juha T. Huiskonen, David I. Stuart, Geoff Sutton, Dennis H. Bamford

**Affiliations:** 1 Division of Structural Biology, University of Oxford, The Henry Wellcome Building for Genomic Medicine Headington, Oxford, UK; 2 Diamond Light Source, Harwell Science and Innovation Campus, Didcot, UK; 3 Molecular and Integrative Biosciences Research Program, Faculty of Biological and Environmental Sciences, University of Helsinki, Helsinki, Finland; 4 Molecular Biophysics and Integrated Bioimaging Division, Lawrence Berkeley National Laboratory, 1 Cyclotron Road, Berkeley, USA; 5 Present Address: Department of Microbiology, Faculty of Agriculture and Forestry, University of Helsinki, Helsinki, Finland; 6 Present Address: Howard Hughes Medical Institute, Janelia Research Campus, Ashburn, VA USA; 7 Laboratory of Structural Biology, Helsinki Institute of Life Science, University of Helsinki, Helsinki, Finland

## Abstract

Viruses are a significant threat to both human health and the economy, and there is an urgent need for novel anti-viral drugs and vaccines. High-resolution viral structures inform our understanding of the virosphere, and inspire novel therapies. Here we present a method of obtaining such structural information that avoids potentially disruptive handling, by collecting diffraction data from intact infected cells. We identify a suitable combination of cell type and virus to accumulate particles in the cells, establish a suitable time point where most cells contain virus condensates and use electron microscopy to demonstrate that these are ordered crystalline arrays of empty capsids. We then use an X-ray free electron laser to provide extremely bright illumination of sub-micron intracellular condensates of bacteriophage phiX174 inside living *Escherichia coli* at room temperature. We have been able to collect low resolution diffraction data. Despite the limited resolution and completeness of these initial data, due to a far from optimal experimental setup, we have used novel methodology to determine a putative space group, unit cell dimensions, particle packing and likely maturation state of the particles.

## Introduction

Whilst viral complexity and size often limits the growth of crystals suitable for classical crystallographic characterisation^1^, recent advances in electron cryo-microscopy (cryo-EM) have opened another route to high resolution structure determination^2–4^. Alternatively, X-ray free electron lasers (XFELs) offer less stringent crystal size requirements; using femtosecond-duration pulses that are over a billion times brighter than synchrotron radiation^5^. This has recently allowed the determination of a high-resolution structure from virus microcrystals^6^.

Although crystals are conventionally grown *in vitro*, the production of protein crystals *in vivo* may be achieved via numerous biological processes^7^. The relatively small size of *in vivo* crystals means that useful diffraction data can be collected only at microfocus synchrotron beamlines or XFELs^8–10^. Collecting diffraction data *in cellulo* minimises potential mechanical damage of fragile crystals and so has the potential for improving the quality of diffraction, however the signal-to-noise of the diffraction data will be adversely affected by the presence of extraneous cellular material^11,12^. Thus, for large unit cells and small crystals the intensities of the Bragg peaks will be dramatically reduced (for instance, similar sized crystals of small picornaviruses will have average intensities hundreds of times less than for lysozyme), whereas the background noise increases in proportion to the amount of extraneous material illuminated. Therefore, very careful experimental design will be needed to obtain useful measured diffraction intensities.

Most bacteriophages terminate an infection cycle by host cell lysis, which limits the accumulation of virus particles within the cell. However, there are some mutant cell lines available which are resistant to lysis by certain bacteriophages. For instance, mutation of the host gene *slyD* (sensitivity to lysis), which encodes peptidyl-prolyl cis-trans isomerase, can block lysis by phiX174, precluding viral escape^13,14^. We have therefore chosen to investigate the structure of phiX174 within infected *E. coli slyD* cells as a proof of principle for what could be developed into a more general vehicle.

**Figure 1:**
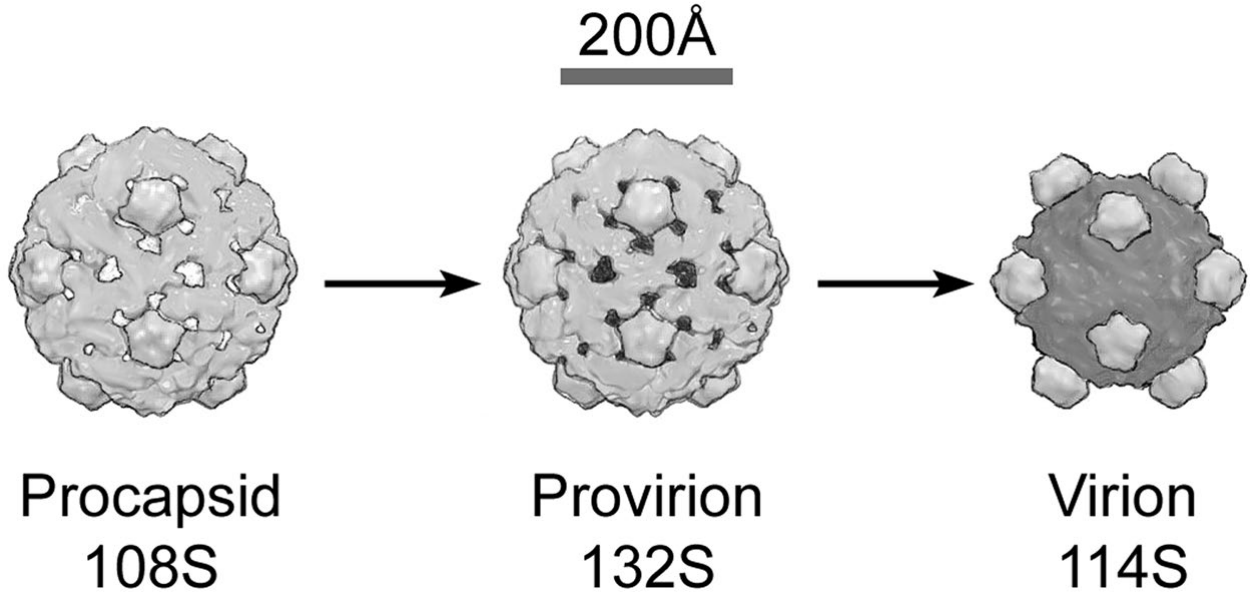
Schematic of the stages in the assembly and maturation of phiX174, a T = 1 bacteriophage. The procapsid (pdb 1cd3^16^) contains proteins B, D, F, G & H and develops into the provirion^20^ by losing at least some of the B proteins and gaining protein J and DNA. Subsequent formation of the mature virion (pdb 2bpa^18^) occurs through the loss of D proteins and the remaining B proteins.

PhiX174′s lifecycle encompasses three distinct states (Fig. 1), all of which are icosahedral and have been studied, *in vitro*, by crystallography^15–19^ and electron microscopy^20,21^. The first stage is assemblage into a procapsid (108 S) form, which, upon loss of the B scaffolding protein, and gain of a J protein, concomitant with packaging of the ssDNA genome, forms the 132 S intermediate provirion, which is of a similar size^16,17,22–24^. Maturation to the virion (114 S) occurs with loss of the D scaffolding protein, and a significant collapse of the structure^22,24^. We report analysis of cells infected with both wild-type (wt) virus and *AmbJ*^−^ mutant virus. The latter are unable to package DNA, since they do not possess the packaging protein, J. Using electron microscopy (EM) and X-ray diffraction, we demonstrate that small crystalline arrays form within cells. The experimental setup-up available, whilst unable to fully explore the potential of the method, nonetheless provided useful information, demonstrating the arrest of PhiX174 maturation at the procapsid state.

## Results

### phiX174 forms crystalline condensates in most cells 4.5 h post infection

**Figure 2:**
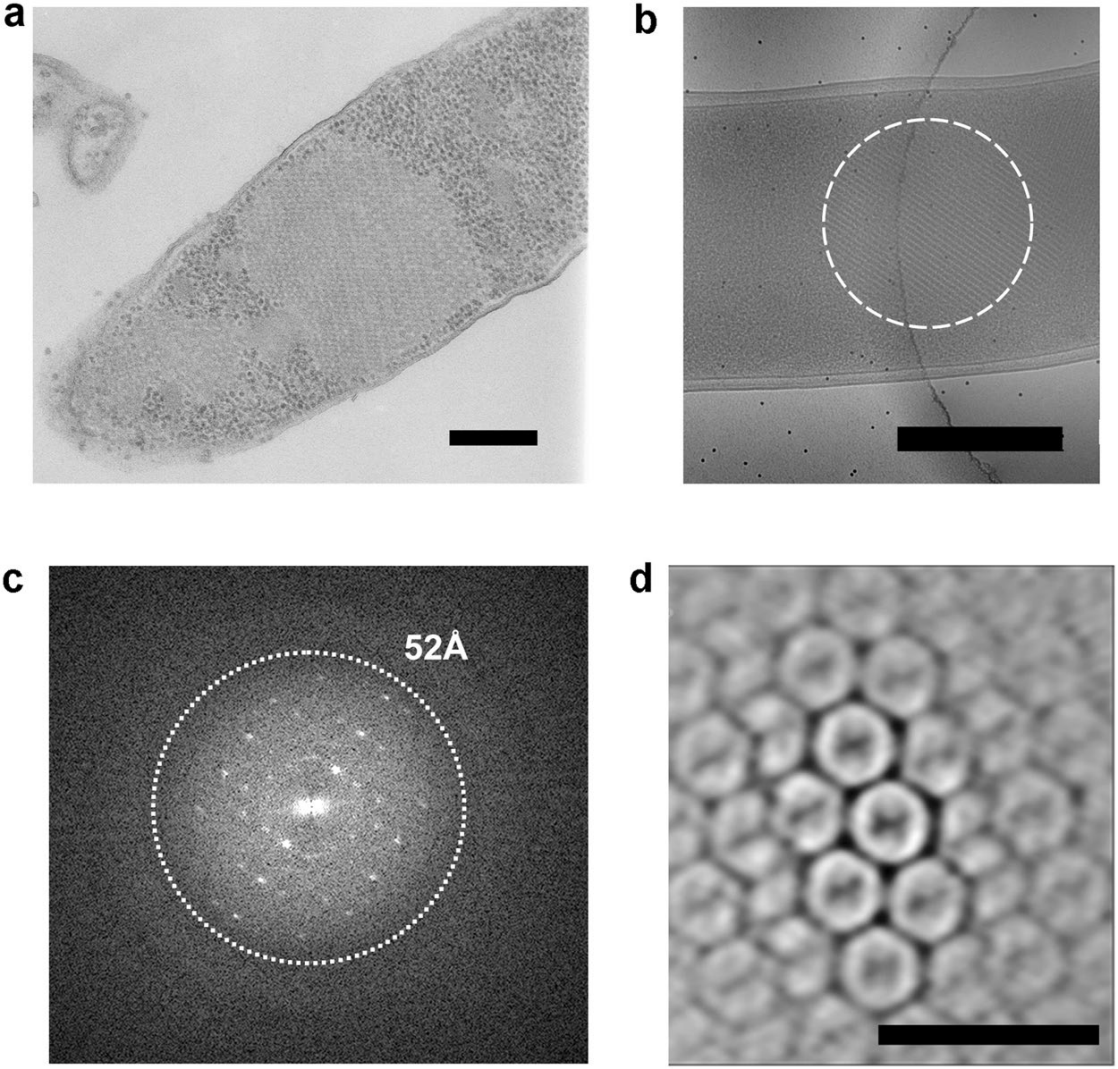
Electron microscopy. (**a**) Ultrathin section of a *E. coli* C990 *slyD1* cell infected with wt phiX174 at 4.5 h p.i. Scale bar shows 200 nm. (**b**) Two-dimensional projection image of similarly infected *E. coli* C990 *slyD1* cell. Scale bar shows 500 nm. (**c**) Fourier transform of the area shown in (**b**). (**d**) Section through averaged tomogram density. Scale bar shows 100 nm.

Thin-section EM of *Escherichia coli* C990 *slyD* infected with both wt and *AmbJ*^−^ phiX174 revealed the presence intracellular viral condensates in lysis-defective conditions^14^ (see Methods), the result of particle accumulation (Fig. 2a). For example, by 1.5 h post infection, aggregates of wt virus particles were seen in some 50% of the cells. By 4.5 h post infection, these had developed so that approximately 75% of cells contained condensates of empty particles, of which roughly one third appeared to be crystalline. We therefore selected 4.5 h post infection as the time point for further analysis.

### Electron Cryo-Microscopy indicates the crystal lattice is reasonably well-ordered

The crystallinity of the crystal-like arrays seen at 4.5 h post infection (for both wt and *AmbJ*^−^ virus) was then visualised using whole-cell three-dimensional tomographic reconstructions and sub-tomogram averaging. The condensates, *circa* 200 × 200 × 200 nm^3^ in volume, nearly filled the cell interior. Power spectra calculated from 2D projection images of cells confirmed that the condensates possessed crystalline order up to a resolution of ~52 Å (Fig. 2b,c). It appears that a major spacing of the lattice is around 340 Å. Sub-tomogram averaging suggested that the capsids were ordered on a lattice and were empty (Fig. 2d).

### XFEL analysis of infected cells

Cells were grown and infected adjacent to the Linac Coherent Light Source (LCLS), at Stanford University. A slurry of 4.5 h post infection cells was pumped through a gas dynamic virtual nozzle (GDVN)^25^ at room temperature into the X-ray beam at the LCLS CXI beamline^5,26^. Diffraction patterns were collected on a CSPAD detector^27^ ~2.5 m from the interaction region of the beam with the jet to record low angle diffraction from condensates.

The majority of data were collected on wt phiX174 at a wavelength of 1.768 Å, while approximately 30% of the dataset was taken from the *ambJ*^−^ mutant. Data for this latter sample were collected at both 1.768 and 1.306 Å wavelengths. At these wavelengths, the maximal resolutions at the detector edge were approximately 46 and 34 Å, respectively.

**Figure 3:**
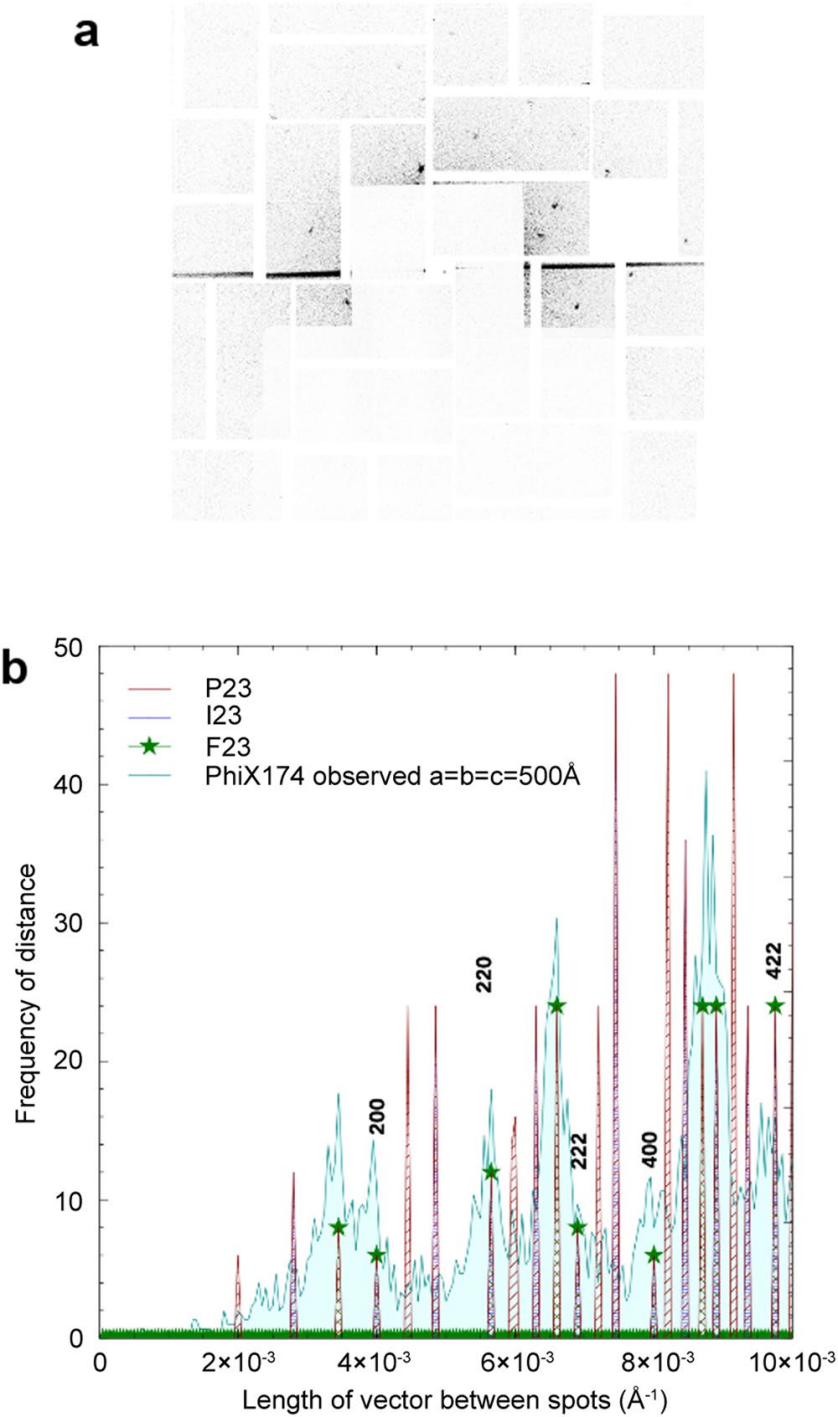
Diffraction data and analysis. (**a**) Example of a strong diffraction pattern from phiX174 wild-type with a clear lattice. Panel shadowing was a consequence of a silicon support used to protect the detector. The black streak was observed on many patterns, although at different angles and is presumably derived from the beam reflecting from the jet edge. (**b**) Histogram of vector distances illustrating selection process of correct cubic space group. Blue fill peaks correspond to combined data from wild type and mutant data sets. Patterned peaks show predicted frequency of vector distances for face- (F), body- (I) and primitive- (P) centred structures. The closest match to our observed data (pale blue fill, with blue outline) is the face-centred cubic space group (green stars).

At the time the experiment was performed the beam and jet were larger than would have been ideal for this experiment. Ideally one would match the beam and the jet to the crystal size, whereas in practice these had diameters of ~2 and 4 μm, respectively. Nevertheless, Bragg diffraction was observed, but limited to ~50 Å spacings (Fig. 3a).

### Initial XFEL Parameters

Based on calibrations from thermolysin data collected shortly before phiX174^28^, the detector distance was estimated to be 2.49 m. Note that this is dependent on accurate estimates of the thermolysin unit cell^29^ and wavelength calibration at LCLS, neither of which were known to a confidence higher than 1%.

The two wavelengths of 1.768 and 1.306 Å were initially derived from LCLS log files. Wavelengths may, however, be taken on a per image basis (Supplementary Fig. 1). The shot-to-shot variation in wavelength is much less that the likely errors in distance, and reprocessing some of the data using these unique per shot energies gave no significant improvement, therefore all further analysis used the overall wavelengths from the log file.

Very low crystal-to-beam hit rates and highly intense artifacts from flaring, a likely consequence of reflection of the incident beam from the edge of the liquid jet (see Fig. 3a), precluded the use of conventional spot finding algorithms even with stringent masking. 168,456 images were initially triaged on the basis of the compressed image size. The extent of jpeg compression is related to information content in the image and we observed that the 3.1 M pixel images compressed to a wide range of sizes (0.124 to 0.421 MB for 2 × 2 binned images). Inspection of a small subset of images revealed that those showing diffraction were <0.3 MB. Using this criteria we were able to immediately eliminate 72% of the images (examination of a substantial subset of these revealed that no diffraction patterns had been missed), whereas the triaged images for the wt sample showed a success rate of 7.2% when screened manually. For the ~680 diffraction patterns identified the coordinates of spots were recorded by hand. Spot positions on the detector were mapped into reciprocal space and pair-wise vectors between all observed spots on each image were calculated. By generating a histogram of these inter-spot distances, a ‘pseudo-powder pattern’ was formed, as described in Ginn 2016^30^ (shown in light blue on Fig. 3b). There was no significant difference in the diffraction between the wt and mutant particles.

Based on the apparent high symmetry indicated by the sparse powder pattern a cubic space group seemed likely (this was consistent with the condensates seen in the cryo-ET reconstructions). In addition, as will emerge in the discussion below, although we strictly need to observe symmetry in the diffraction intensities to confirm the cubic space group, a cubic space group is consistent with elements of the inherent symmetry of the virus particle and the alignment of particle and crystal symmetry elements is a common feature of most virus crystals. For simplicity, we therefore assume that this is the case here. Thus, a systematic fitting of allowed face-centred (F), body-centred (I) and primitive- (P) space groups was performed (Fig. 3b). The powder pattern facilitates the identification of centering operations, since successively more reflections are systematically absent for P, I and F lattices. For a primitive lattice, all reflections can be of non-zero intensity (shown in red in Fig. 3b). For a body centered lattice the sum of the three components of the indices must be even (shown with dark blue hatching) for non-zero intensity and for face centering all three pair-wise sums of the components must be even (shown with green hatching and a star). This produced a convincing best fit for a face-centred cubic space group, with an approximate cell edge of 500 Å (see Supplementary Fig. 2). Assuming a cubic lattice this limits the possible space groups which can accommodate a chiral virus particle to F*23*, F*432* and F*4*_*1*_*32*. The highest subgroup of icosahedral symmetry that can be accommodated in a crystal lattice is 23 symmetry. All of the putative space groups contain points of 23 symmetry, F*23* has four such points whilst F*432* and F*4*_*1*_*32* each have eight. Even with the most economical packing, only F*23* can accommodate a particle of the size of phiX174 on a point of 23 symmetry, since four, but not eight, particles can be accommodated in a cell of this size (detailed packing arguments discussed below). We therefore conclude that the most likely space group of the viral arrays grown in infected cells is F*23*, with a = b = c = ~500 Å. Note that purified viral particles and procapsids have been crystallised previously, however none of these crystals are isomorphous with those grown in infected cells (the calculated powder patterns agree poorly, Supplementary Fig. 3).

### Putative Maturation State

Since the diffraction from the wt and *AmbJ*^−^ was indistinguishable and protein J is required for genomic packaging, it seemed likely that our sample is that of a procapsid (furthermore in electron micrographs the condensates appeared to be empty particles, Fig. 2). It is still possible that the condensates represent the mature virion, but in an empty form. Spontaneous maturation in some viral procapsids has been observed *in vitro*^16,31–33^, and most notably, for the homologous bacteriophage G4 (sequence identity of 40–66%), where both conformational change, and external scaffolding led to the formation of an empty ‘mature virion’^34^.

Knowing the space group and unit cell dimension, we can calculate the separation of the centres of neighbouring virus particles (*e.g*. at 0, 0, 0 and 0, ½, ½) as ~353 Å. From published data, the minimum/maximum diameters of the virion and procapsid are 300/342 and 348/352 Å respectively (Table 1). This strongly suggests that the in-cell crystals contain procapsids. Although there are some experimental uncertainties, the true unit cell dimension will lie well within the range of 480 to 520 Å (which correspond to the extreme distances and wavelengths within the experimental uncertainty). Within this range, the only sensible packing for either a procapsid or mature virus is for the procapsid with a cell dimension close to 500 Å, thus in the best packing for a mature virion (480 Å cell) the closest approach of sidechains is about 20 Å (Supplementary Fig. 4).

**Table 1:** Summary published crystal data for phiX174.

Property	Maturation State	
	Procapsid	Mature virus
*PDB Code*	1cd3^16^	2bpa^18^
*Space Group*	I2_1_3	P2_1_
*Unit cell dimensions*		
*a, b, c/Å;*	774.00, 774.00, 774.0	305.58, 360.78, 299.4
*α, ß, γ/°*	90.00, 90.00, 90.00	90.00, 92.89, 90.00
*Outer Radius/Å*	176	171
*Outer Diameter/Å*	352	342
*Outer Spherical Volume/Å* ^3^	22, 836, 345.90	20, 944, 834.90

## Discussion

We have established a routine procedure to produce cells, 75% of which contain phiX174 viral condensates. We have also demonstrated that these intracellular viral condensates form ordered sub-micron crystals that are capable of diffracting X-rays to produce informative patterns at XFELs. This represents a proof of principle for the extension of XFEL crystallography to systems with orders of magnitude lower signal than previously reported, and lends promise to further exploration of generic methods for viral structural analysis to bypass traditional crystallisation and handling. This holds potential for difficult-to-crystallise viral targets.

There is a clear route to improving the signal-to-noise for the diffraction data. At the time the experiments were performed, the beam and jet were larger than ideal. Assuming that the beam could be trimmed to the maximal crystal cross-section within the X-ray beam (~0.4 × 0.4 μm^2^ supposing maximum crystal dimensions are approximately twice those observed on average by EM), compared to the beam used here (which intersects approximately 2 × 2 μm^2^ of jet) a 25-fold improvement in signal-to-noise could be obtained. Furthermore, if the jet could be thinned from ~4 μm to the dimensions of a cell, ~1 μm, the total potential gain would be ~100-fold. Although the bacterial cells will scatter somewhat more strongly than the liquid jet, this is probably not an unreasonable estimate. Finally, focussing the entire beam onto the crystal would increase the signal ~25-fold and the signal-to-noise by a further factor of five. Although extraordinarily challenging with current liquid jet technology, since the hit-rate will decline precipitously, solid-phase supports, offering high speed and precision, may be used instead to recover much of this potential 500-fold gain^35,36^. A significant improvement in hit rate may also be achieved with the recently reported Acoustic Droplet Ejection technology^37^.

If it is possible to extend the applicability of this method, and render it a generic vehicle for icosahedral, and perhaps any non-pleomorphic, virus particles, *in cellulo* crystallisation might provide new targets that may only be crystallised within intracellular conditions. It remains to be seen whether such advances provide a method competitive with electron microscopy for large complexes, but at the very least they may offer a synergistic tool.

## Methods

### Sample preparation

Cultures were grown in Luria broth. Lysis-defective cells of logarithmic *Escherichia coli* C990 *slyD1* cultures were infected with phix174 using a multiplicity of infection of 10 and CaCl_2_ and MgCl_2_ were added to final concentrations of 5 and 10 mM, respectively. After infection, cells were incubated for 5 min without aeration and then aerobically at 37 °C. For thin-section EM, cells were fixed 4.5 h post infection (p.i.) with 3% (v/v) glutaraldehyde and sections were prepared as previously described^38^. The micrographs were taken with JEOL1200EX electron microscope operating at 80 kV.

### Electron cryo-microscopy and tomography

For electron cryo-microscopy and tomography, unfixed infected cells and 10 nm gold clusters were added to an electron microscopy grid 4.5 h p.i. and the grid was plunge-frozen in a liquid ethane-propane mixture. Data were collected on a Tecnai Polara electron microscope (Thermo Fischer) operating at 300 kV at liquid nitrogen temperature and equipped with a 4k × 4k CCD camera (Gatan) mounted behind an energy filter (Gatan) operating at zero-loss mode (20 eV slit). For two-dimensional imaging, a series of 40 images (total dose of 80 *e*^−^Å^−2^) was acquired at 12 µm under focus and images were computationally aligned in IMOD^39^. Images were high-pass filtered to 10000 Å^−1^ to remove low frequency features arising from variations in sample thickness. For three-dimensional tomographic reconstruction of viral aggregates, a tilt series was collected from −60° to +60° in 4° increments at 12 µm under focus, with a maximum dose of 70 *e*^*−*^Å^−2^ in SerialEM^40^ and reconstructed in IMOD using the gold clusters as fiducial markers. Template matching and sub-tomogram averaging of ordered condensate regions was performed in Jsubtomo^41,42^, taking into account the missing wedge of tomographic reconstructions. Averages were low pass filtered to 80 Å^−1^ for visualization.

### Serial femtosecond crystallography

Diffraction data were collected at the Linac Coherent Light Source (LCLS) of SLAC National Accelerator Laboratory (Menlo Park, CA, USA) on the Coherent X-ray Imaging (CXI) beamline^26^. Intact cells were injected at room temperature using a Gas Dynamic Virtual Nozzle^25^ at a flow rate of 50 µl *min*^−1^. X-ray pulses were of 50 fs duration with 2.0 × 10^12^ photons per pulse and a beam diameter of c.a. 2 μm. In order to calculate the X-ray wavelength, electron energies were converted to photon energies, based on the undulator parameter K^43^.

Femtosecond diffraction snapshots were recorded at 120 Hz on the downstream position Cornell-SLAC pixel array (CSPAD) detector^27^ at a sample-detector distance estimated to be 2.49 m.

### Pseudo-powder pattern generation

Approximately 2000 images with diffraction were derived from the output streams. Diffraction spots were then manually selected from these diffraction images and the corresponding pixel coordinates recorded (Ginn, unpublished software). Vectors between pairs of spots were generated using cppxfel^30^, and a histogram showing the frequency of the occurrence of the lengths of these vectors was termed the “pseudo-powder pattern”. This method has been described previously^44^.

### Crystal symmetry determination and packing analysis

Packing of the procapsid (PDB 1cd3)^16^ and mature capsid (PDB 2bpa)^18^ asymmetric units was visualised using PyMOL^45^.

## Electronic supplementary material

Supplementary Information
