# Supplementary material for: Towards in cellulo virus crystallography
Source: Sci Rep. 2018 Feb 28;8:3771. doi: 10.1038/s41598-018-21693-3 (PMC5830620; doi:10.1038/s41598-018-21693-3)
Supplement: Supplementary file 1 — Supplementary Information [file 41598_2018_21693_MOESM1_ESM.docx]

**Supplementary information for:**

Towards *in cellulo* virus crystallography

Helen M. E. Duyvesteyn, Helen M. Ginn, Maija K. Pietilä, Armin Wagner, Johan Hattne, Jonathan M. Grimes, Elina Hirvonen, Gwyndaf Evans, Marie-Laure Parsy, Nicholas K. Sauter, Aaron S. Brewster, Juha T. Huiskonen, David I. Stuart, Geoff Sutton, Dennis H. Bamford

**Contains**

**Supplementary Figures 1 – 4**


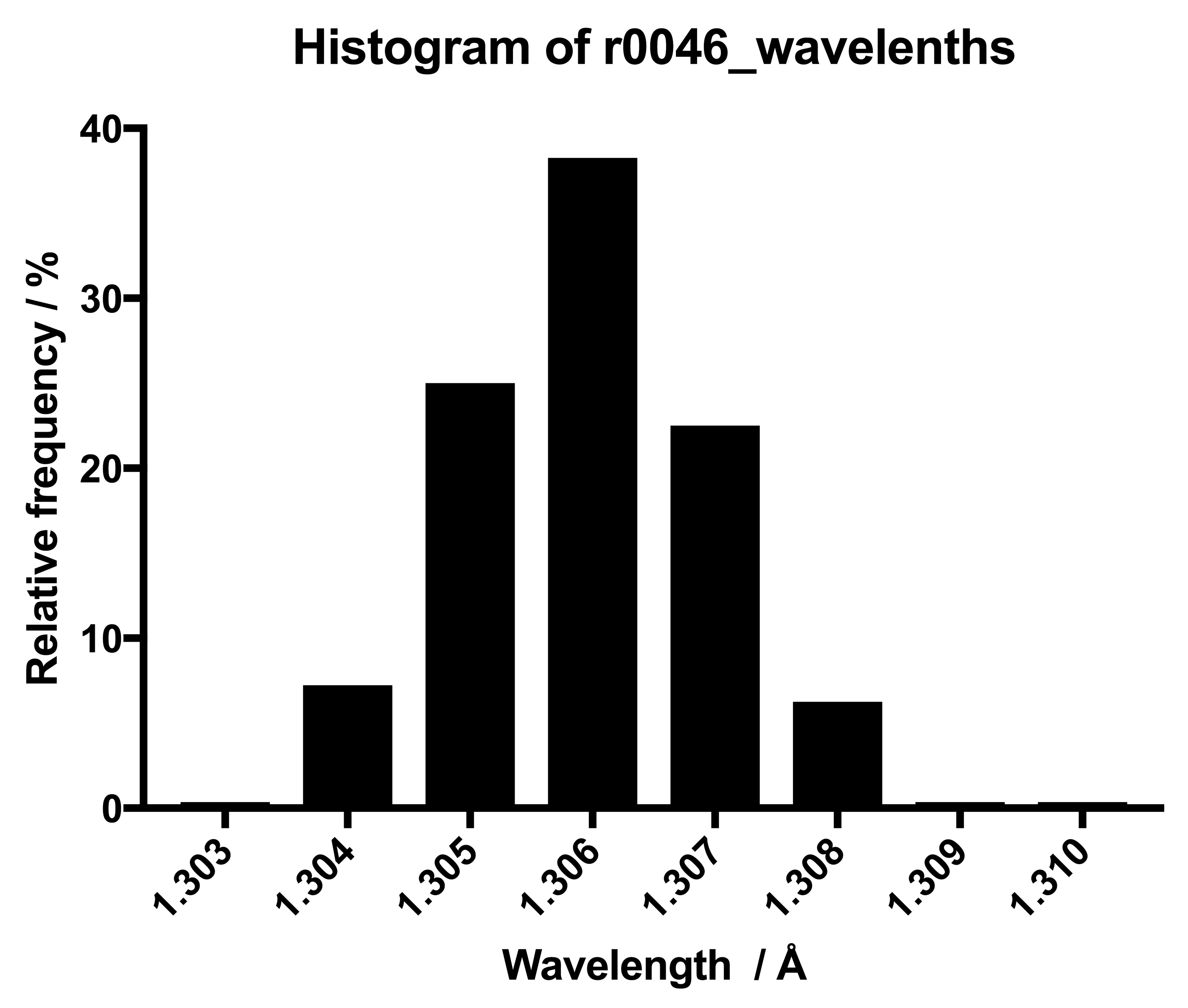

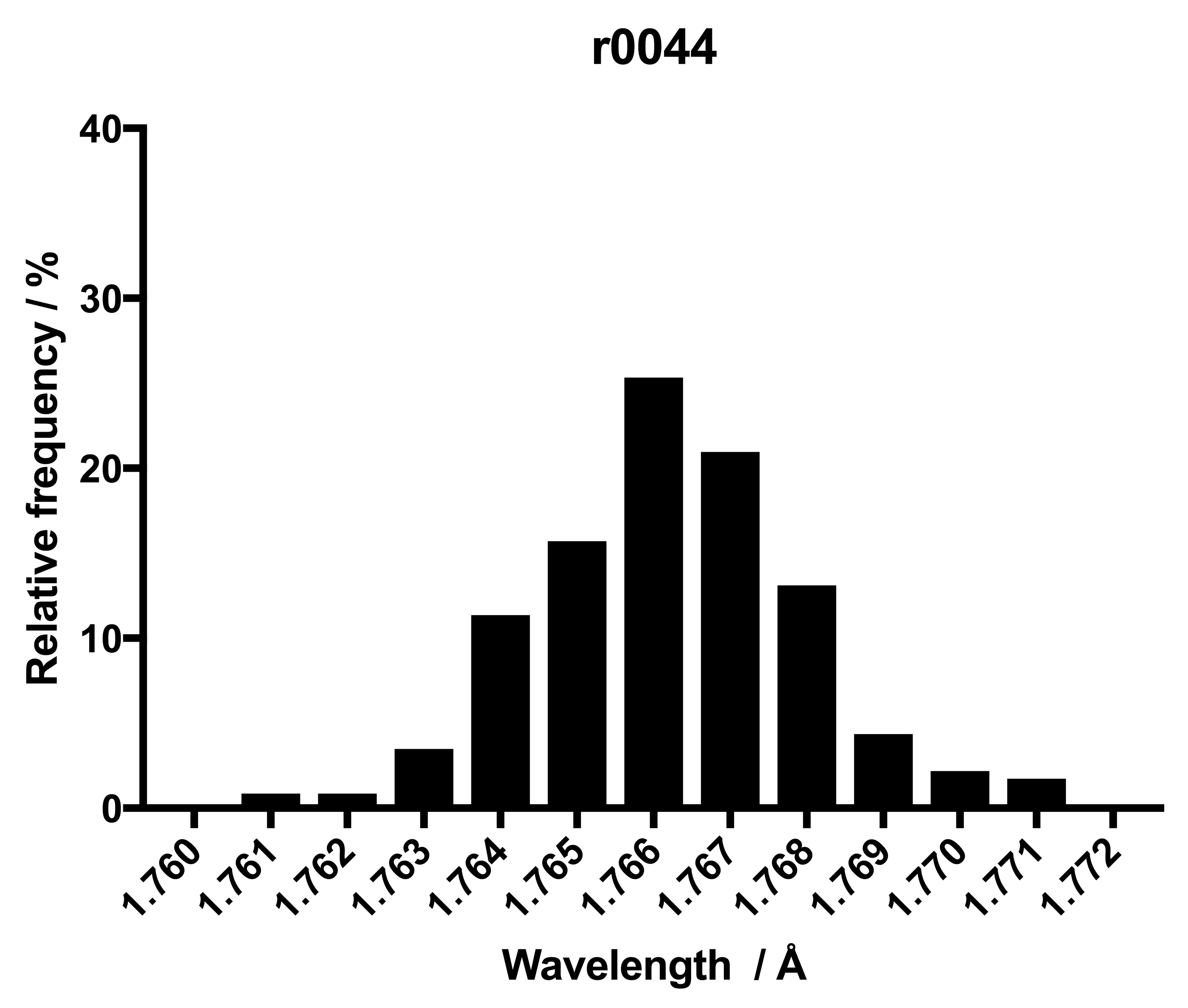

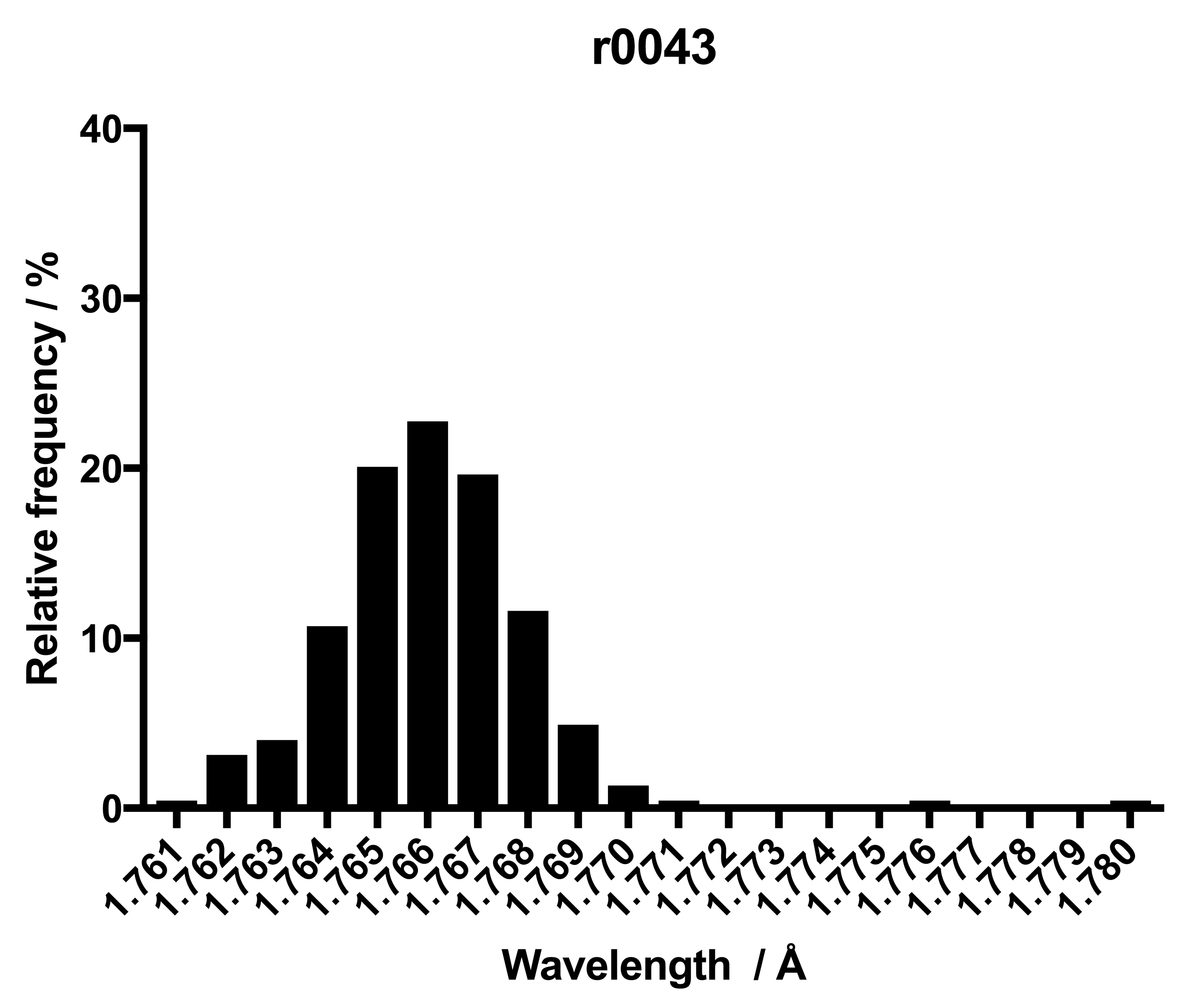

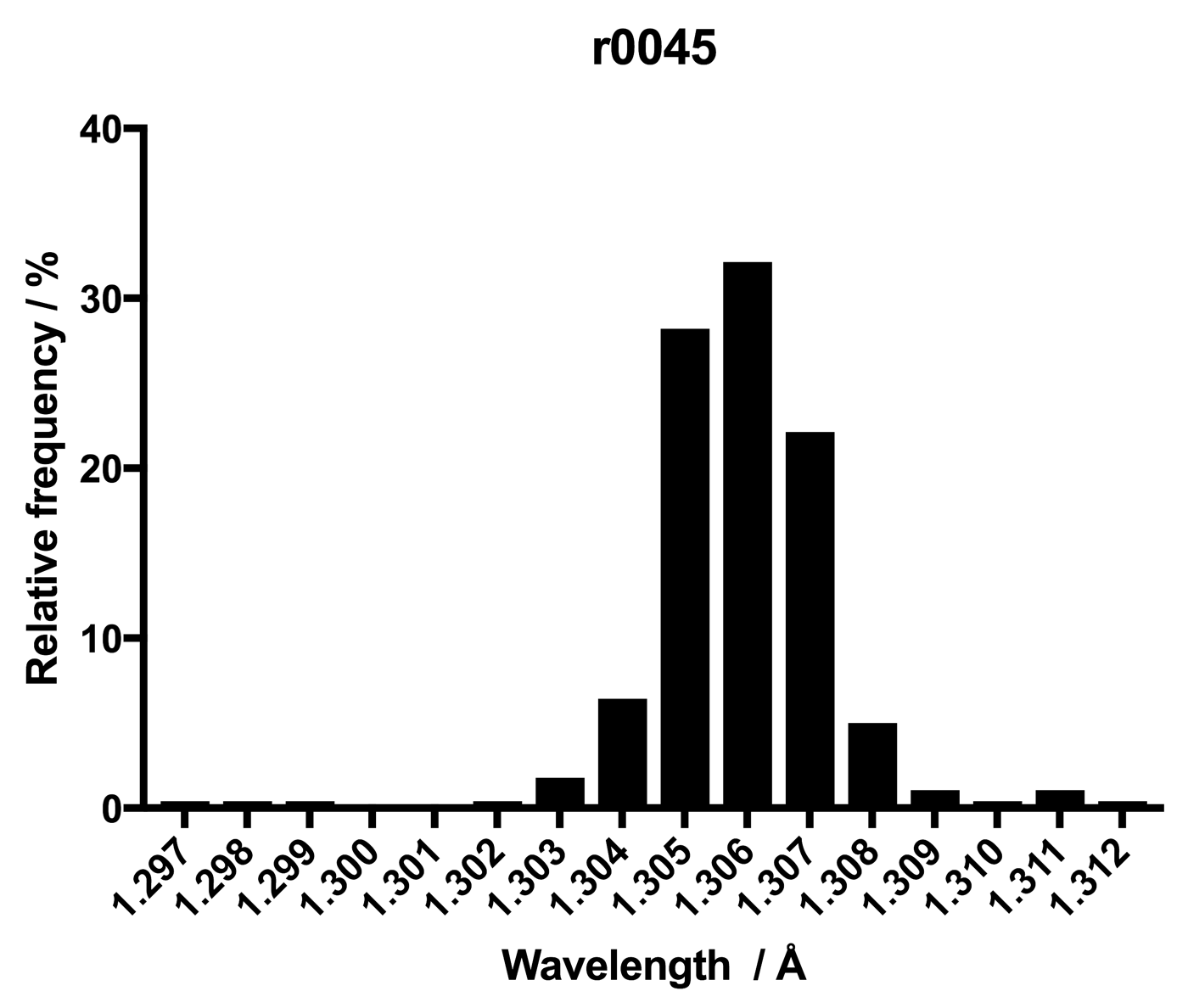


**Supplementary Figure 1.** Distribution of photon wavelengths per shot, inferred from XFEL data files. Clockwise from top: run 43, 44 (longer wavelengths), 46, 45 (shorter wavelength). The respective arithmetic mean photon wavelengths for the two wavelengths employed was determined as 1.768 Å (top), and 1.306 Å (bottom).

**Supplementary Figure 2.** Pseudo powder pattern analysis of cell size for wild type (left) and *AmbJ^-^* mutant (right) diffraction data. Blue fill shows observed data, while red peaks show the anticipated vector lengths in Å (i.e. vector distances) based on the stated unit cell dimensions.

**
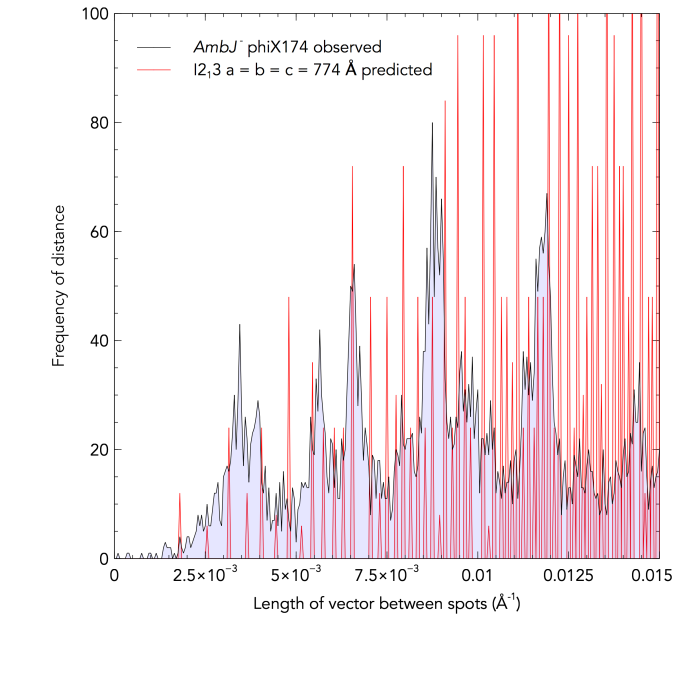
**
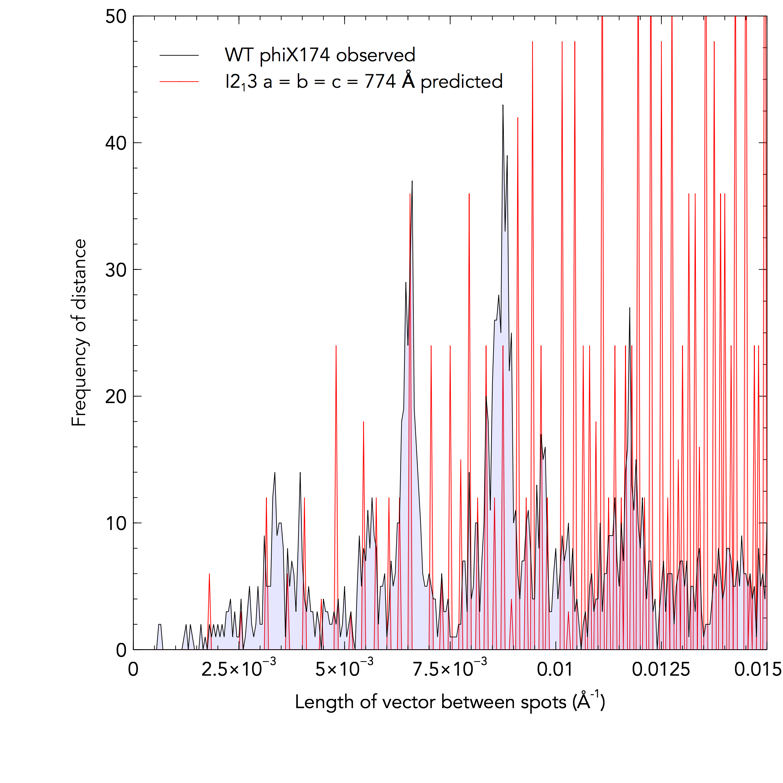


**
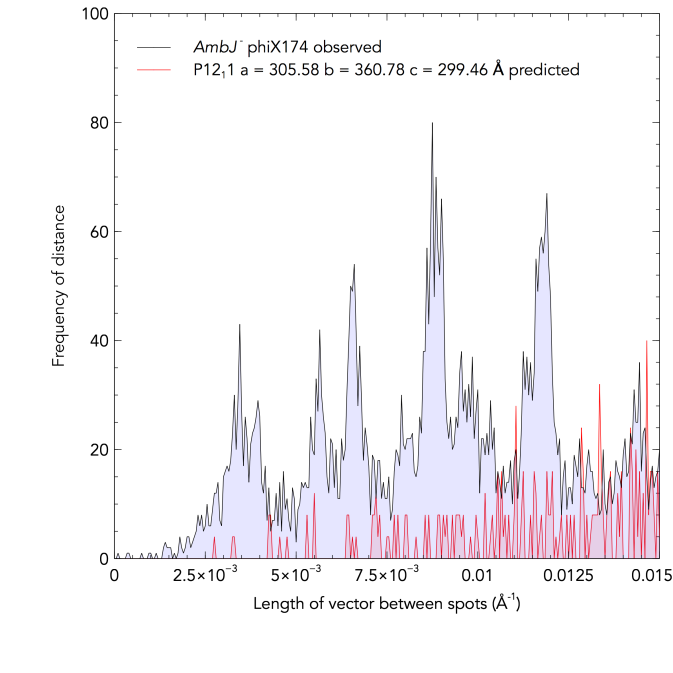
**
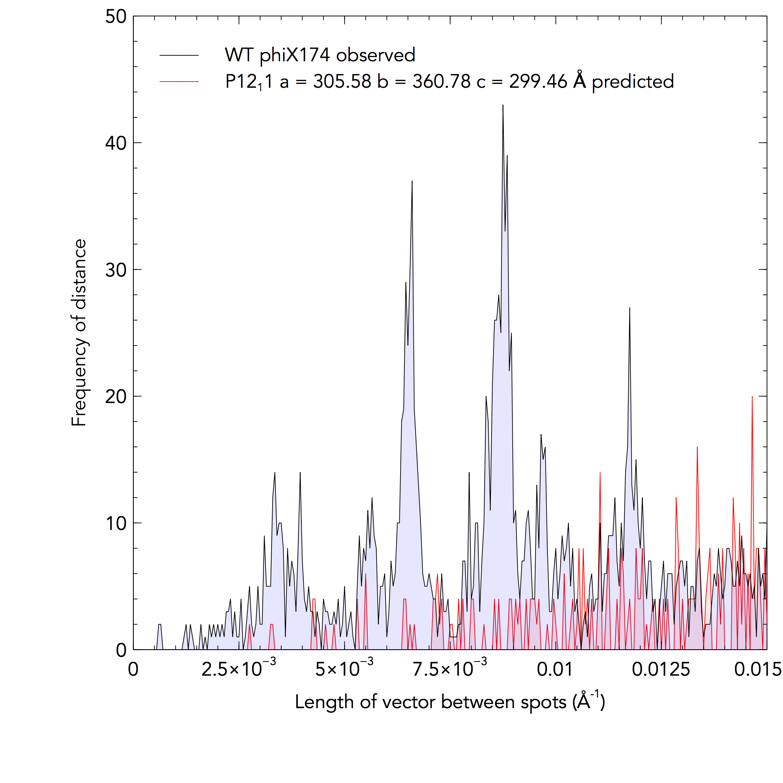


**Supplementary Figure 3.** Pseudo powder pattern analysis, comparison with published crystal forms. The results show fitting of the different unit cell dimensions for wild type (left) and *AmbJ^-^* mutant (right) to the diffraction data. Blue fill peaks correspond to observed data, while red peaks show the anticipated vector lengths based on the stated unit cell dimensions. Top space group and unit cell from procapsid (PDB 1cd3) and bottom for the mature virus (PDB 2bpa).

**
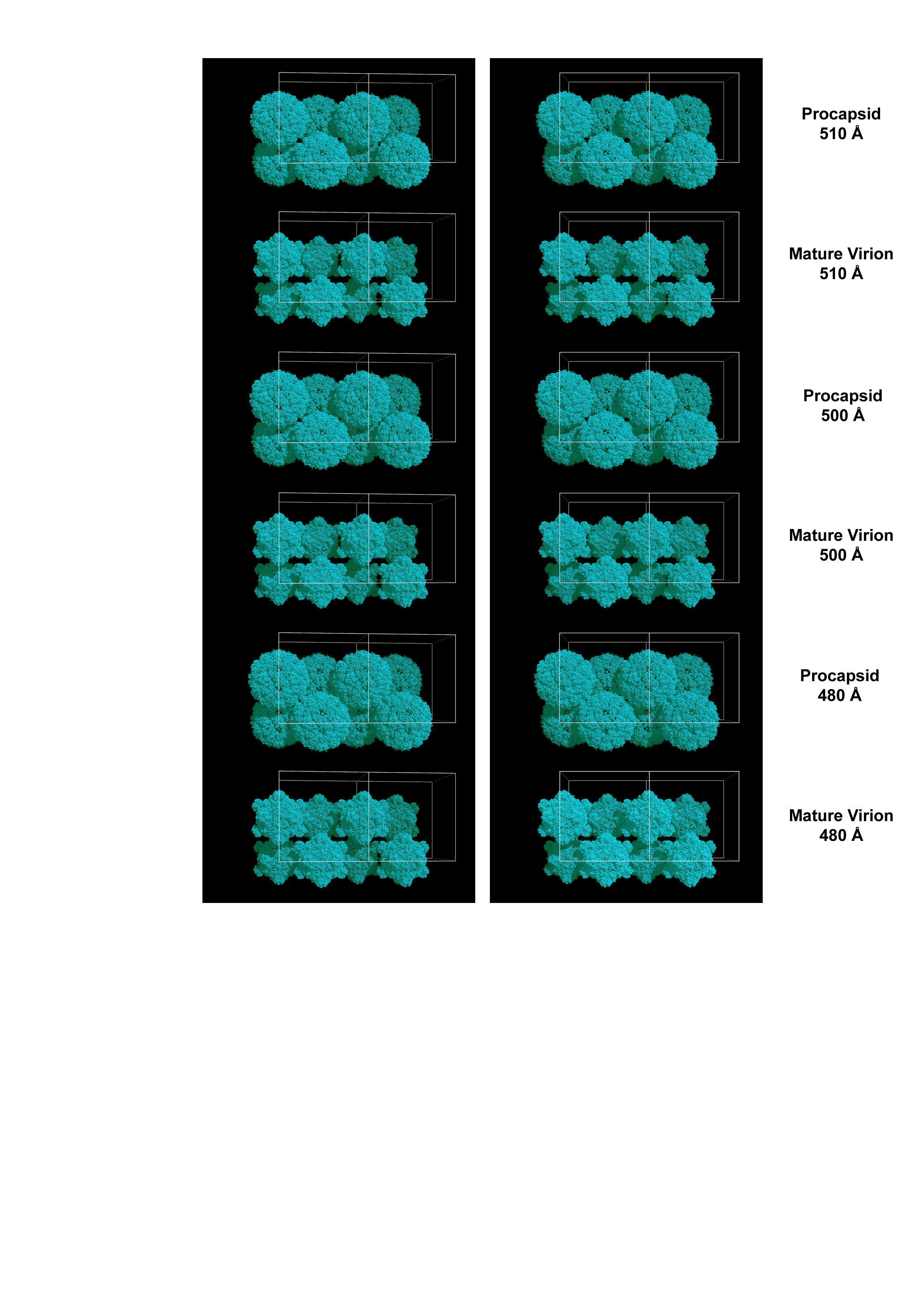
**

**Supplementary Figure 4.** Wall-eye stereo representations of packing within a F*23* unit cell with differing unit cell dimensions. C-alpha atoms only are shown, and so the particles will be slightly larger, with the 500 Å cell giving excellent packing for the procapsid. Alternate images show procapsid (PDB: 1cd3) and mature virion (PDB: 2bpa). Top two rows a = 510 Å, next two 500 Å, bottom two rows 480 Å.
